# Hemodynamic Responses in Lower Limb Lymphedema Patients Undergoing Physical Therapy

**DOI:** 10.3390/biology10070642

**Published:** 2021-07-10

**Authors:** Bianca Brix, Olivier White, Christian Ure, Gert Apich, Paul Simon, Andreas Roessler, Nandu Goswami

**Affiliations:** 1 Gravitational Physiology and Medicine Research Unit, Division of Physiology, Otto Loewi Research Center, Medical University of Graz, 8010 Graz, Austria; bianca.brix@medunigraz.at (B.B.); paul.simon@stud.medunigraz.at (P.S.); andreas.roessler@medunigraz.at (A.R.); 2 Unit 1093, Cognition, Action and Sensorimotor Plasticity, Institut National de la Santé et de la Recherche Médicale, 21000 Dijon, France; olivier.white@u-bourgogne.fr; 3 Clinical Center for Lymphatic Disorders, Wolfsberg State Hospital, KABEG, 9400 Wolfsberg, Austria; Christian.ure@kabeg.at (C.U.); gert.apich@kabeg.at (G.A.); 4 Physical Medicine and General Rehabilitation, KABEG, Wolfsberg Site, 9400 Wolfsberg, Austria

**Keywords:** lymphedema therapy, cardiovascular responses, sit-to-stand test, orthostatic loading, orthostatic hypotension, lymphatic drainage

## Abstract

**Simple Summary:**

Different stimuli can influence posture in daily life and, therefore, impose a risk of a solid balance during standing. To maintain this upright position, the capacity to recognize changes in posture and react appropriately is essential. The increased frequency of falls owing to postural hypotension, along with a lack of postural stability, is a serious problem not just in the elderly, but also different patient groups (e.g., cardiac patients, diabetic patients, or patients recovering from stroke). Similar changes in blood pressure regulation, as well as cardio-postural control, might occur in patients suffering from lymphedema due to differences in fluid volumes of the lower limbs. Lymphedema therapy could therefore affect hemodynamic responses during orthostatic loading (sit-to-stand test) due to the variable amounts of fluid in the lower limbs throughout therapy. To our knowledge, no previous study has ever longitudinally investigated the inter-relationship between lymphedema and hemodynamic responses and changes in volume regulatory hormones during orthostatic loading over three weeks of lymphedema treatment. We report here that lymphedema patients did not show signs of orthostatic hypotension, demonstrating that those patients do not seem to be at an increased risk for orthostatic intolerance and falls. However, lymphedema treatment showed to have a potential beneficial effect on cardiovascular responses during orthostatic challenge (sit-to-stand test) in lymphedema patients.

**Abstract:**

Background: Lymphedema arises due to a malfunction of the lymphatic system, leading to extensive tissue swelling. Complete decongestive therapy (CDT), which is a physical therapy lasting for 3 weeks and includes manual lymphatic drainages (MLD), leads to fluid mobilization and increases in plasma volume. Here, we investigated hemodynamic responses induced by these fluid shifts due to CDT and MLD. Methods: Hemodynamic parameters were assessed continuously during a sit-to-stand test (5 min baseline, 5 min of standing, and 5 min of recovery). This intervention was repeated on days 1, 2, 7, 14, and 21 of CDT, before and after MLD. Volume regulatory hormones were assessed in plasma samples. Results: A total number of 13 patients took part in this investigation. Resting diastolic blood pressure significantly decreased over three weeks of CDT (*p* = 0.048). No changes in baseline values were shown due to MLD. However, MLD led to a significant decrease in heart rate during orthostatic loading over all epochs on therapy day 14, as well as day 21. Volume regulatory hormones did not show changes over lymphedema therapy. Conclusion: We did not observe any signs of orthostatic hypotension at rest, as well as during to CDT, indicating that lymphedema patients do not display an elevated risk of orthostatic intolerance. Although baseline hemodynamics were not affected, MLD has shown to have potential beneficial effects on hemodynamic responses to a sit-to-stand test in patients undergoing lymphedema therapy.

## 1. Introduction

The effects of gravity on the cardiovascular system while standing up create a dip in blood pressure. This can be explained by the fact that blood is pooled in the lower extremities. Consequences of this can be the loss of consciousness (syncope) if the body cannot compensate this blood pressure drop upon standing up [1,2]. Orthostasis can have a huge impact in persons with a known record of dizziness upon standing up or in patients in general (e.g., cardiac patients, diabetic patients, or patients recovering from stroke) [3]. In those people, the cardiovascular system is not able to maintain the mean arterial pressure at a required level when standing up (orthostatic challenge), further leading to a critical decrease in cerebral blood flow, eventually resulting in fainting. The causes of syncope are multiple; it possibly arises due to cardiac issues, hormonal factors, cerebral autoregulation, or autonomic dysfunction [4]. To maintain a stable posture, the capacity to recognize changes in posture and respond appropriately is required [5,6].

The lymphatic vessels transport several liters of lymphatic fluid throughout the whole body every day [7,8,9]. As a result, any malfunction in this system might result in fluid accumulation within the tissue [10]. A chronic and progressive disease, known as lymphedema, may develop [10,11]. Characteristic for this disease are severe tissue swellings, usually occurring in the upper or lower extremities due to a stasis in lymph flow. This fluid accumulation can reach up to 5–15 L [12]. Complete decongestive therapy (CDT) is a noninvasive, multicomponent physical treatment over three weeks that is usually applied to reduce swelling [13]. The physical treatment consists primarily of manual lymphatic drainage (MLD) and compression, as well as bandaging of the swollen body part, along with additional aerobic movements that provide leverage over the swelling. This type of nonoperative, conservative treatment is known to have a preventative, instead of curative, character [14].

In lymphedema patients, cardio-postural control might be affected due to different fluid volumes in the lower limbs, pre- and post-therapy, as well as due to plasma volume changes during physical therapy [10,13,15,16]. However, to our knowledge, there is no previous study focusing on investigating orthostatic intolerance during lymphedema therapy. We have previously reported that three weeks of physical therapy leads to fluid shifts in the limbs, as well as the whole body, in lower limb lymphedema patients [13]. Lymphedema therapy could therefore affect hemodynamic responses during orthostatic loading due to different amounts of fluid in the lower limbs, pre- and post-therapy. Increases of plasma volume due to MLD, as we have previously reported [13], might influence volume regulating hormones. Thus, we investigated hemodynamics at rest and in response to orthostatic loading before, during, and after CDT. We hypothesize that three weeks of physical therapy will have beneficial effects on hemodynamics at rest. We further hypothesize that hemodynamic responses to a sit-to-stand test (orthostatic loading) will be modulated due to CDT and/or MLD over three weeks. As far as we are aware, no previous study has ever investigated the inter-relationship between lymphedema and hemodynamic responses and changes in volume regulatory hormones during orthostatic loading longitudinally over three weeks of lymphedema treatment.

## 2. Materials and Methods

This investigation was performed at the clinical center for lymphatic disorders, KABEG, Wolfsberg, Austria. Data analysis was conducted at the Division of Physiology at the Medical University of Graz, Austria. Prior to commencing the study, a positive ethical vote was received from the responsible Ethics Committee of Carinthia (A 03/17), as well as from the Ethics Committee at the Medical University of Graz (29-090 ex 16/17). The study was conducted following the latest Declaration of Helsinki (2013). Each patient was given extensive information about the procedures, and provided both informed and written consent. The signed consent forms are stored at the Medical University of Graz.

### 2.1. Patients

Male and female patients diagnosed with stage II primary and secondary lower limb lymphedema were recruited directly at the clinical center for lymphatic disorders. Diagnosis was performed according to the recommendations reported in the most recent consensus document provided by the International Society of Lymphology (ISL) [17]. Enrollment of patients into the study followed distinct criteria. Included were patients with lymphedema of the lower extremities at stage II prior to undergoing three weeks of CDT. The following exclusion criteria were applied: patients that showed any clinical signs of mental disorders, patients taking specific medications that could possibly influence the assessed variables (e.g., diuretics or beta-blockers), as well as patients with a medical history of cardiovascular diseases e.g., syncope. Pregnant women, as well as patients who received CDT in the last 12 months, were also not allowed to participate.

### 2.2. Therapy Protocol

The treatment protocol was based on the recommendations by Döller [18,19] and is routinely performed at the clinical center for lymphatic disorders in Wolfsberg. Briefly, the three weeks of lymphedema therapy (CDT) comprised of 30 min of manual lymphatic drainages (MLD), aiming at mobilizing and eliminating edema fluid. This was performed every weekday (5 times per week). MLD was always performed on the same time of the day by different therapists. Then, the application of compression bandages to the lower limbs were worn during the day and overnight. Whilst wearing these compression garments, patients also performed physical exercise, such as walking and/or ergometry, for about 60 min each day, either individually or in groups.

### 2.3. Assessment of Hemodynamic Responses (Sit-to-Stand Test)

**Figure 1:**
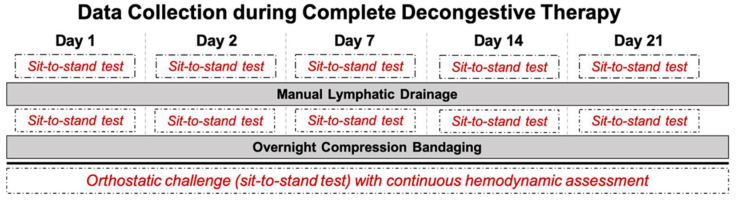
Detailed overview of data collection during complete decongestive therapy (CDT), as well as manual lymphatic drainage (MLD), over three weeks of lymphedema treatment. Sit-to-stand test was performed twice per day, before and after MLD, as indicated in the dashed boxes.

All measurements were performed in the morning (from 8:00 am to noon) in a quiet and slightly dimmed room. Room temperature was set between 21 and 24 °C. Humidity was controlled and remained in the range of 50–60%. Hemodynamic assessment was performed at different timepoints over three weeks of CDT (on day 1, day 2, day 7, day 14, and day 21) to investigate a time-course effect of CDT. Patients were asked to refrain from coffee 24 h before the assessment, but were allowed to have breakfast 1 h prior to the measurement. Further, to determine the effect of MLD, the measurements were carried out immediately before and after 30 min of MLD on each of the indicated days, as further depicted in Figure 1. To assess hemodynamic parameters at baseline (rest) after a 10-min rest period, as well as in response to orthostatic loading, patients were asked to perform a sit-to-stand test, which consists of three parts: (A) 5 min sitting as baseline; (B) 5 min standing; (C) 5 min sitting again as recovery. Throughout all three stages, a Task Force Monitor^®^ (CNSystems, Graz, Austria) was utilized to continually assess hemodynamic parameters noninvasively. The following parameters were continuously recorded: heart rate (HR), systolic and diastolic blood pressure (SBP/DBP), as well as mean arterial pressure (MAP). For further details, please see Trozic et al. 2019 [2].

### 2.4. Assessment of Volume Regulating Hormones

Aldosterone and plasma renin activity (PRA) were assessed via a commercially available ELISA kit (Hölzel Diagnostika, Cologne, Germany). Atrial natriuretic peptide (ANP) was assessed via an RIA kit without prior extraction (Nichols Institute, San Juan Capistrano, CA, USA). Arginine vasopressin (AVP) was assessed via an ELISA kit (Biomedica, Vienna, Austria). A commercially available ELISA kit (Cloud-Clone Corp., Katy, TX, USA) was used to measure adrenomedullin (ADM). Pro-brain natriuretic peptide (proBNP) was assessed via a competitive inhibition-enzyme-linked immunosorbent assay (Cloud-Clone Corp., Katy, TX, USA). All hormone assessments were performed at the Medical University of Graz, Austria.

### 2.5. Data Analysis and Statistics

**Figure 2:**
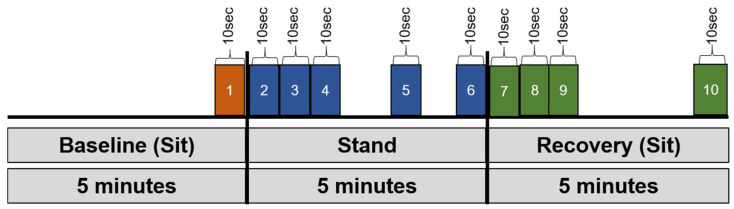
Overview of the epochs that we defined and used for data analysis. Several key epochs were examined (191): (1) final 10 s of sitting baseline, (2) 0–10 s, (3) 10–20 s, (4) 20–30 s, (5) 290–300 s, and (6) final 10 s of stand phase, as well as (7) initial 0–10 s, (8) 10–20 s, (9) 20–30 s, and (10) final 10 s of sitting recovery.

MATLAB Software (Version 2016b, The MathWorks Inc., Natick, MA, USA) was used to analyze hemodynamic data as previously described in Trozic et al., [18]. Epochs of 10-s intervals (average of time series) were analyzed from each phase of the sit-to-stand test, as depicted in Figure 2. Because of the scarcity of complete datasets, two-way ANOVA for repeated measurements was used to examine hemodynamic responses, using the following variables for each measurement day separately: epochs and pre-/post-MLD. Level of significance was fixed at *p*-values < 0.05. SPSS Statistics (Vers. 23, IBM, Armonk, NY, USA) was utilized for statistical analysis. Figures were generated via GraphPad Prism (Vers. 8.4.1, GraphPad Software Inc., San Diego, CA, USA). All data are displayed as means ± standard deviation (SD). Figures depict means ± standard error of the mean in order to provide better visualization.

## 3. Results

In total, 13 patients (10 females and 3 males, 57 ± 8 of age, 167.2 ± 8.3 cm height, 91.0 ± 23.5 kg weight) were included. Demographic details are depicted in Table 1.

### 3.1. Baseline Hemodynamic Parameters

**Figure 3:**
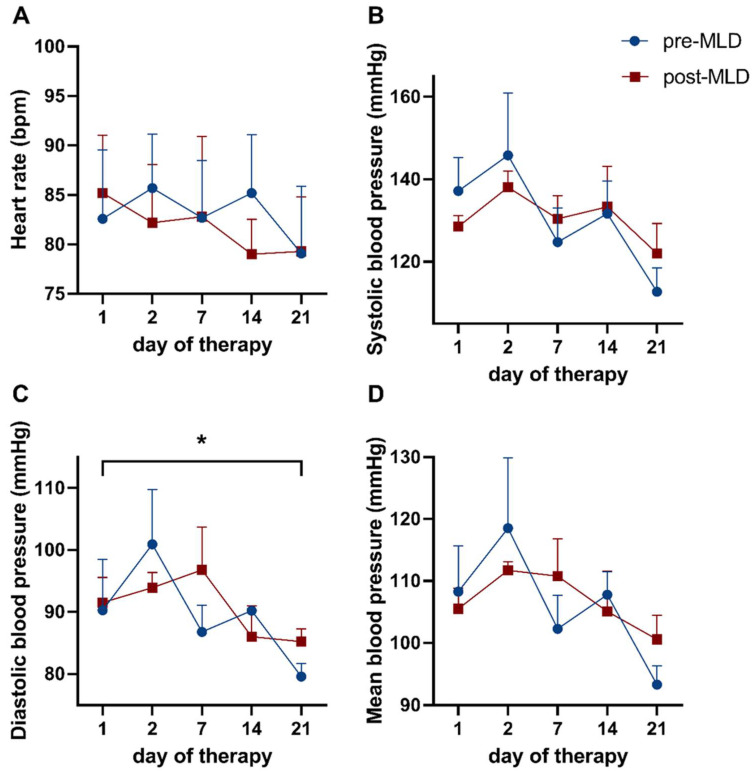
Time course showing baseline hemodynamic parameters over three weeks of complete decongestive therapy (CDT), before and after manual lymphatic drainage (MLD). Displayed are (**A**) heart rate, as well as (**B**) systolic, (**C**) diastolic, and (**D**) mean blood pressure. Data before MLD are depicted as blue dots; values after MLD are shown as red circles. Asterisks mark significant differences (*). Note: ticks on the *x*-axis (day of therapy) are not spaced equally in time.

Resting diastolic blood pressure (DBP) (epoch 1) significantly reduced from 90.9 ± 10.6 mmHg before to 82.4 ± 4.0 mmHg (F_(4,12)_ = 3.31; *p* = 0.048) after three weeks of CDT (Figure 3C, Table 2). Baseline values in heart rate (HR), systolic blood pressure, as well as mean blood pressure (MBP) were not altered (F_(4,16)_ = 1.17; *p* = 0.362; F_(4,12)_ = 2.65; *p* = 0.085 and F_(4,12)_ = 2.89; *p* = 0.069, respectively) as a result of three weeks of CDT (Figure 3A,B,D).

Manual lymphatic drainage did not affect HR (F_(1,4)_ = 1.17; *p* = 0.699), SBP (F_(1,3)_ = 0.00; *p* = 0.998), DBP (F_(1,3)_ = 2.28, *p* = 0.632), and MBP (F_(1,3)_ = 0.07; *p* = 0.815), as no significant changes could be observed in these parameters (Figure 3A–D).

### 3.2. Responses in Hemodynamic Parameters during Orthostatic Loading

**Figure 4:**
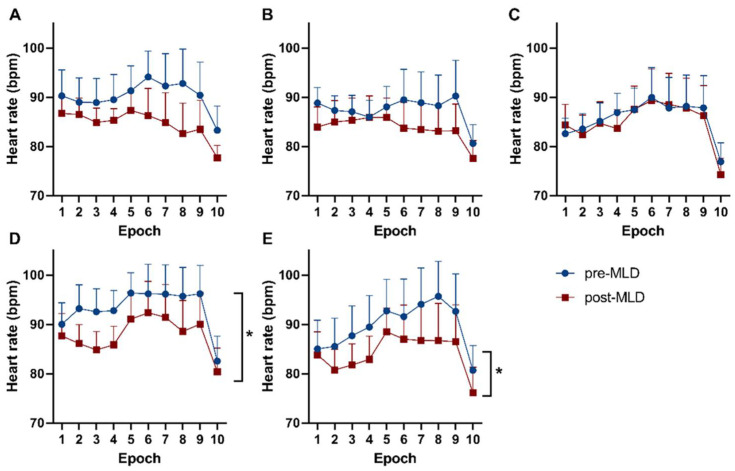
Responses of heart rate during distinct epochs of a sit-to-stand test throughout Table 1. (**A**) day 1, (**B**) day 2, (**C**) day 7, (**D**) day 14, and (**E**) day 21 of lymphedema therapy. The blue dots show data before MLD. Red boxes reflect data after MLD. Significant differences in heart rate pre- vs. post-MLD are labeled with asterisks (*).

Heart rate significantly reduced over all indicated epochs on therapy day 14, as well as therapy day 21 (F_(1,9)_ = 6.07; *p* = 0.036 and F_(1,9)_ = 5.24; *p* = 0.048, respectively), due to 30 min of MLD (Figure 4). An overall effect over all epochs was observed in heart rate values during orthostatic loading on the first (F_(9,72)_ = 12.53; *p* = 0.014), seventh (F_(9,81)_ = 6.56; *p* < 0.001), fourteenth (F_(2,21)_ = 5.14; *p* < 0.001), and twenty-first (F_(2,19 )_= 6.67; *p* = 0.006) day of CDT. Responses in blood pressure did not show any changes during orthostatic loading pre- compared to post-MLD on each individual measurement day.

## 4. Discussion

Our results show that diastolic blood pressure at rest reduced during three weeks of lymphedema therapy. However, no significant changes were observed in all other baseline hemodynamic parameters resulting from physical treatment. Hemodynamic responses showed a significantly reduced heart rate response to orthostatic loading assessed via a sit-to-stand test towards the end of CDT (after 2 weeks).

Over three weeks of CDT, diastolic blood pressure at rest significantly decreased from 90.9 ± 10.6 mmHg to 82.4 ± 4.0 mmHg (*p* = 0.048). As we are not aware of any previously published study reporting alterations in blood pressure due to lymphedema therapy, this is a novel finding. It is unclear if the drop of diastolic blood pressure was caused by lymphedema therapy or exercise done as part of full decongestive therapy, as physical activity and exercise is one aspect of lymphedema therapy, along with MLD and compression bandages. A total of 30–60 min of exercise a week has been shown in studies to result in a significant reduction in diastolic blood pressure in critical hypertensives [20]. In this study, neither daily pedometer steps nor daily workout time were reported. However, since CDT was administered in a hospital setting with tight time constraints, rehabilitation sessions, and workout classes, the daily exercise duration was similar for all participants, about 60 min per day.

No changes in baseline hemodynamic parameters could be observed after MLD. Previous studies examining cardiovascular and hemodynamic changes due to physical therapy report inconclusive outcomes [21,22,23]. Esmer et al. 2019 revealed immediate changes in blood pressure, as well as heart rate, following one session of manual lymphatic draining in several different body regions [21]. They reported a decrease in systolic and diastolic blood pressure directly after MLD, which was performed in the lower extremities [21]. This is in contradiction to the results we present here. Another study performed by Kim et al. 2009 showed that heart rate variability significantly changed between the group receiving MLD and the control group [24]. However, this was reported in healthy participants and not in lymphedema patients. Hemodynamic parameters (heart rate and blood pressure) did not show changes after MLD in the patients that were included in this study. Our findings are also supported by a study performed by Ramos et al. 2015. They reported that no changes in blood pressure occurred during and post a single application of 30 min of MLD [22]. Leduc et al. 2011 used echocardiography and investigated changes in cardiac parameters during and due to lymphatic drainage. In their study, the patients showed a significant reduction in heart rate, whereas blood pressure did not change due to MLD [23]. A potential explanation for the inconclusive findings in the different studies could be the patients that were included. Some studies included healthy participants [21,22], while, in others, patients with heart disease participated [23]. In our study, hemodynamic changes due to MLD were determined in lower limb lymphedema patients. This has not been reported previously.

Another hypothesis was that lymphedema patients will display signs of orthostatic hypotension. This hypothesis is based on the increased fluid volumes in the legs, before vs. after physical therapy. Further, we have previously reported an elevation of 1.5% of plasma volume post-MLD [13]. The sit-to-stand test was successfully completed by all patients. Further, lymphedema patients did not show any signs of orthostatic hypotension. The definition of orthostatic hypotension is a reduction of systolic blood pressure levels of 20 mmHg and/or a reduction in diastolic blood pressure by 10 mmHg during upright posture [2,25]. This did was not observed in lymphedema patients, neither at the beginning (baseline), nor over the three weeks of treatment. Our findings indicate that lymphedema patients do not seem to be at an increased risk of orthostatic intolerance. Further, those patients are not at a higher risk of falling.

Furthermore, a significant reduction in heart rate was seen due to MLD over all 10 epochs on the 14th and 21st day of CDT (*p* = 0.036 and *p* = 0.048, respectively). An average reduction in heart rate of 4 bpm was seen on the 14th day and a decrease of 5 bpm on the 21st day of CDT over all epochs of the indicated measurements. Therefore, MLD appears to have possible beneficial effects on cardiovascular parameters/hemodynamic responses to an orthostatic challenge in patients with lymphedema.

Both CDT and MLD did not influence blood pressure responses during orthostatic loading. Finally, volume regulatory hormones were not affected by lymphedema therapy. The amount of fluid shifts due to MLD were not sufficient to induce volume regulatory responses.

### Limitations

One potential weakness of this clinical investigation could be the limited number of patients that could be included (*n* = 13). However, we did perform a sample size calculation a priori, which revealed that an estimated total of 13 patients would be a sufficient sample size to show statistical significance. Moreover, the main strength of this investigation is that each individual patient counted as his/her own control. Therefore, each individual patient was followed over the entire time-course of three weeks of complete physical therapy. The same assessments were conducted over several time points in the same patient. Due to the limited number of patients involved in this study, a separate analysis depending on the different therapists who applied MLD to the patients was not possible. Therefore, future studies should also consider the effect of MLD performed by different therapists. Further, larger cohort studies should be performed in future, which will allow the investigation of different stages and disease durations of lymphedema, distinct etiologies (primary or secondary), in various body parts (upper and/or lower limbs), or by differing therapies (surgery, physiotherapies, or medications).

## 5. Conclusions

Our results show that patients with lymphedema are not at an elevated risk of orthostatic intolerance and falls due to hemodynamic changes, since no signs of orthostatic hypotension during an orthostatic challenge (sit-to-stand test) were observed in those patients at baseline, as well as over three weeks of complete decongestive therapy. Baseline hemodynamic parameters were not affected by manual lymphatic drainage. Nevertheless, lymphatic drainage has a possible beneficial effect on hemodynamic parameters during an orthostatic challenge in patients suffering from lymphedema who are undergoing physical therapy.

## Figures and Tables

**Table 1:** Patients’ demographic details.

ID	Sex	Age (years)	Height (cm)	Weight (kg)	Type of Lymphedema
1	female	55	167	61.0	Secondary bilateral lymphedema
2	male	63	180	116.0	Secondary bilateral lymphedema
3	female	53	178	116.8	Secondary unilateral lymphedema
4	female	52	165	94.7	Secondary bilateral lymphedema
5	female	46	158	62.9	Secondary bilateral lymphedema
6	female	55	159	101.4	Primary bilateral lymphedema
7	male	56	180	90.2	Secondary bilateral lymphedema
8	female	71	159	70.0	Primary bilateral lymphedema
9	female	64	165	103.2	Secondary unilateral lymphedema
10	male	59	176	137.1	Primary unilateral lymphedema
11	female	40	157	73.7	Primary bilateral lymphedema
12	female	66	168	59.9	Primary bilateral lymphedema
13	female	55	161	95.5	Secondary bilateral lymphedema
Mean		57	167.2	91.0	
SD		8	8.3	23.5	

**Table 2:** Changes in resting hemodynamic parameters (baseline values) during three weeks of complete decongestive therapy, pre- vs. post-manual lymphatic drainage (MLD). The data are displayed as means ± standard deviation.

Characteristic		Complete Decongestive Therapy					
		Day 1	Day 2	Day 7	Day 14	Day 21	Estimated Marginal Means
Heart rate (bpm) *n* = 5	Before MLD	82.6 ± 15.6	85.7 ± 12.2	82.7 ± 12.9	85.2 ± 13.2	79.1 ± 15.2	83.1 ± 13.5
	After MLD	85.2 ± 13.1	82.2 ± 13.2	82.8 ± 18.2	79.0 ± 7.9	79.3 ± 12.4	81.7 ± 11.8
	Estimated marginal means	83.9 ± 13.8	84.0 ± 11.6	82.7 ± 15.5	82.1 ± 9.2	79.2 ± 12.4	
Systolic blood pressure (mmHg) *n* = 4	Before MLD	137.2 ± 16.4	145.8 ± 30.3	124.8 ± 16.5	131.7 ± 15.9	112.7 ± 11.5	130.4 ± 12.9
	After MLD	128.5 ± 5.4	138.1 ± 7.8	130.4 ± 11.1	133.3 ± 19.6	122.0 ± 14.5	130.4 ± 6.8
	Estimated marginal means	132.8 ± 10.2	142.0 ± 18.4	127.6 ± 13.4	132.5 ± 13.1	117.3 ± 12.4	
Diastolic blood pressure (mmHg) *n* = 4	Before MLD	90.3 ± 16.4	100.9 ± 17.7	86.8 ± 8.7	90.2 ± 1.2	79.6 ± 4.3	89.6 ± 7.4
	After MLD	91.5 ± 8.2	93.9 ± 4.9	96.8 ± 13.9	86.0 ± 10.0	85.2 ± 4.1	90.7 ± 5.6
	Estimated marginal means	90.9 ± 10.6	97.4 ± 7.5	91.8 ± 11.3	88.1 ± 5.2	82.4 ± 4.0	
Mean blood pressure (mmHg) *n* = 4	Before MLD	108.3 ± 14.8	118.5 ± 22.8	102.3 ± 10.9	107.8 ± 7.4	93.3 ± 6.1	106.1 ± 9.4
	After MLD	105.5 ± 6.7	111.7 ± 2.8	110.8 ± 12.1	105.1 ± 13.0	100.6 ± 7.8	106.8 ± 5.3
	Estimated marginal means	106.9 ± 9.5	115.1 ± 12.3	106.6 ± 11.2	106.4 ± 8.5	97.0 ± 6.6	

## Data Availability

The data presented in this study are available on request from the corresponding author.
